# Deltoid contracture: A study of nineteen cases

**DOI:** 10.4103/0019-5413.40256

**Published:** 2008

**Authors:** Debabrata Banerji, Chinmay De, Ananda K Pal, Sunil K Das, Soumya Ghosh, Shijin Dharmadevan

**Affiliations:** Department of Orthopedics, Burdwan Medical College, Burdwan, West Bengal, India

**Keywords:** Deltoid contracture, surgical release of deltoid contracture, eitopathogenesis of deltoid contracture

## Abstract

**Objective:**

Deltoid contracture is not uncommon in India. Contractures of deltoid often do not have definite etiology. We have critically analyzed the condition as regards the etiopathogenesis and its surgical results.

**Materials and Methods:**

Nineteen patients with deltoid contracture operated between June 1990 and September 2001 were enrolled for a unicentric retrospective study. The surgery was indicated in patients with abduction deformity of more than 30° at the shoulder. The etiology of deltoid contracture was idiopathic (*n* = 13) intramuscular injection in deltoid muscle (*n* = 5) and blunt trauma (*n* = 1). All were operated by distal release (incision near the insertion of the deltoid muscle). The average follow-up was of 9.5 years (range 6-17 years). They were evaluated based on parameters like pain, persistence of deformity, range of shoulder movements and strength of deltoid.

**Results:**

All patients recovered painless full range of shoulder motion except one. The correction of deformity was achieved in all patients and there was no loss of strength of deltoid compared to the opposite side. Histology of excised tissue showed features of chronic inflammation. The complications observed were hypertrophic scar (*n* = 1), painful terminal restriction of shoulder movements (*n* = 1) and prominent vertebral border of scapula (*n* = 1).

**Conclusion:**

Deltoid contracture has features of chronic inflammation, and the intramuscular deltoid injection is the most incriminating factor in its etiopathogenesis. The condition can be effectively managed surgically by distal release of the deltoid muscle combined with excision of the muscular fibrotic contracture band.

## INTRODUCTION

Contracture of deltoid muscle, though uncommon in developed countries,^1^,^2^ is much more common in India. Often it is associated with intramuscular injection in deltoid^3^,^4^ and may be seen among siblings^5^ or certain segregated patriarchal ethnic groups.^3^ The etiology of deltoid contracture is unknown but predisposing factors like intra muscular injections have been described.^3^–^5^ It presents with cosmetic deformity of winging of scapula. The condition is not adequately reported in world literature. Often the diagnosis is missed. The present study aimed to analyzes the etiopathogenesis of deltoid contracture and evaluate the results of surgical treatment.

## MATERIALS AND METHODS

**Figures 1:**
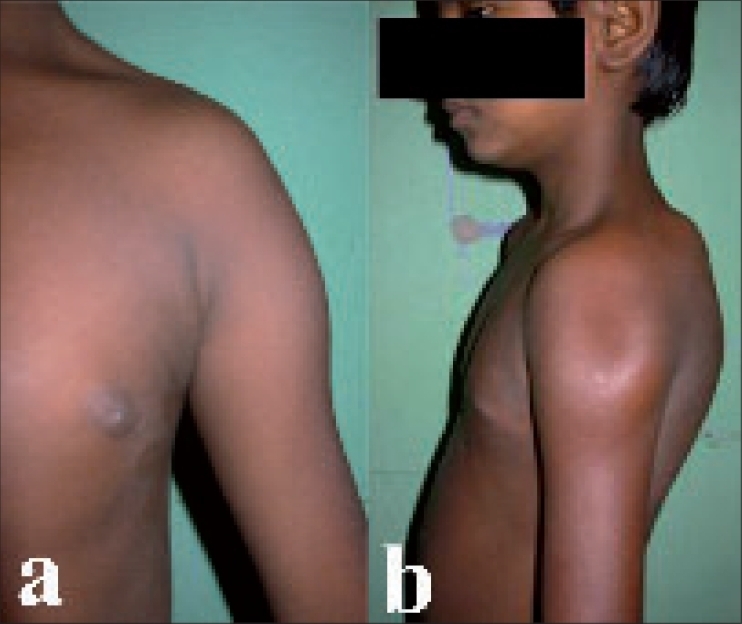
Preoperative clinical photographs of Case 3 showing abduction contracture of shoulder (a), with visible furrow overlying the contracture (b). The radiographs of this patient were normal

From June 1990 to September 2001, 19 patients presented with a deltoid contracture. Eight patients were in the age group of 6-10 years and 11 patients were between 11 and 19 years (12 patients were male and seven females). Fourteen patients had unilateral affection of which 13 had contracture on the left side. Both sides were affected in five and in four of them the left side was severely contracted. Hence the left side was predominantly affected. All patients presented with abduction deformity of shoulder of more than 30° [Figure 1A] persisting for 6-11.5 years duration (average 8.5 years); one had history of blunt trauma and five had a history of intramuscular injection in the deltoid in childhood. Two patients in the series were brother and sister.

On palpation, all patients had palpable contracture band in the lateral segment of the deltoid, of which four had additional contracture bands extending up to the posterolateral segment of the deltoid. Four patients had visible furrow over the contracture band [Figure 1] which helped in their easy identification. These four patients had anterior subluxation of humeral head.

**Figure 2:**
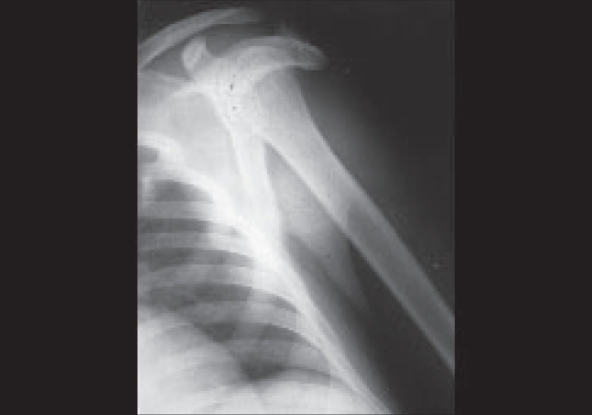
Skiagram of an adult patient (Case 17) with long standing deltoid contracture showing hanging deformity of acromion with anterior subluxation

The abduction deformity in our patients ranged from 30 to 50° with further full abduction possible in all the patients [Table 1]. All the patients presented with a cosmetic deformity of winging of the scapula. On attempted passive adduction at the shoulder, the scapula became unusually prominent. Apart from deformity, pain in the neck, shoulder and upper arm was the most common symptom. There was no secondary deformity of the spine or chest wall. There was no contracture seen in other parts of the body. Skiagrams of the shoulder revealed some typical features like tapering of the clavicle and drooping of acromion covering the greater tuberosity of the humerus, maldevelopment of shoulder joint [Figure 2]. Anterior subluxation of the humeral head was noted in five patients who had contraction bands for more than 10 years. Abduction deformity of more than 30° at the affected shoulder was an indication for surgical treatment in our cases.

**Table 1:** Clinical details of patients

Case	Age (year)	Sex	Side	Etiology	Type of contracture	Abduction deformity (in degrees)
1	10	M	L	Idiopathic	Lateral	35
2	15	M	BL	Idiopathic	Lateral	30
3	9	F	L	Idiopathic	Lateral with posterolateral extension	50
4	11	M	BL	Injection fibrosis	Lateral	40
5	6	F	L	Blunt trauma	Lateral	30
6	14	F	BL	Idiopathic	Lateral	45
7	17	M	L	Idiopathic	Lateral	50
8	6	M	L	Injection fibrosis	Lateral with posterolateral extension	45
9	8	M	L	Idiopathic	Lateral	30
10	12	F	L	Injection fibrosis	Lateral	35
11	10	F	BL	Idiopathic	Lateral with posterolateral extension	40
12	8	M	L	Idiopathic	Lateral	40
13	19	M	L	Injection fibrosis	Lateral	45
14	15	M	L	Idiopathic	Lateral with posterolateral extension	35
15	12	F	L	Idiopathic	Lateral with posterolateral extension	50
16	6	M	BL	Idiopathic	Lateral	30
17	19	F	L	Idiopathic	Lateral	40
18	12	M	L	Injection fibrosis	Lateral	35
19	14	M	L	Idiopathic	Lateral	30

M-male, F-female, L-left, R-right, BL-bilateral

### Operative procedure

**Figures 3:**
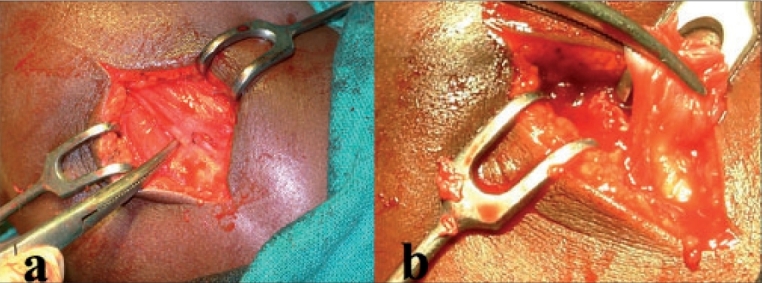
Peroperative radiographs showing delineation of contracture band (a) and excision of the primary contracture band (b)

After general anesthesia patient was positioned supine with a folded sheet under the affected shoulder. The band was approached either by transverse incision (in nine patients) or longitudinal incision (in 10 patients) over the contracture band. The contracture band was then identified and separated from muscle mass completely [Figure 3a]. Distal release, i.e. excision of the band near the insertion of the muscle, was performed in all except one (Case 14), in which case a proximal release was necessary because of the associated subluxation of the humeral head with contracture of joint capsule. Transfixation stitches, were placed through the proximal and distal ends of the clinically palpable ends of the contracted band to prevent bleeding. The band was then excised between the transfixation stitches [Figure 3b]. Excised tissue was sent for histopathological study. Apart from the main band, sometimes it was necessary to divide a few accessory bands. These accessory bands were present within the posterior part of the muscle, which were identified by adducting the shoulder which made them more prominent. Hemostasis was secured meticulously. Postoperatively the affected limb was strapped to the body in full internal rotation and adduction of shoulder till the stitches were removed on the 10^th^ postoperative day. Active shoulder movements were then allowed as soon as the patient was able to perform them within the limits of pain.

## RESULTS

**Figure 4:**
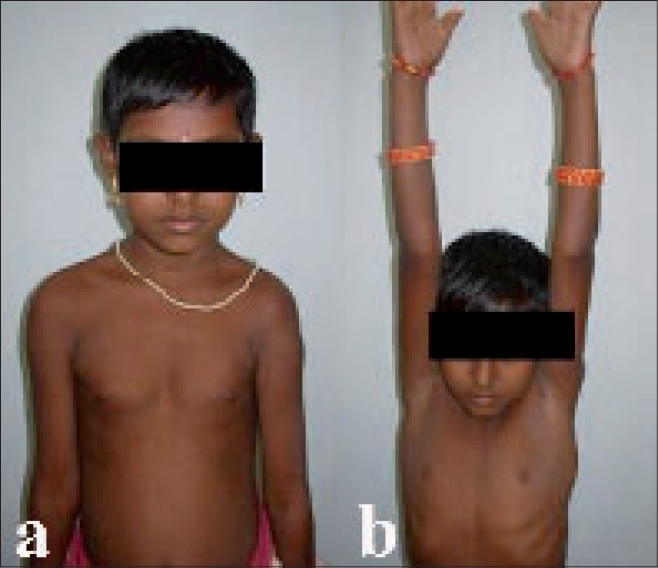
Five-year postoperative clinical photographs of Case 3 showing correction of abduction deformity (a), with satisfactory recovery of shoulder abduction (b)

**Figure 5:**
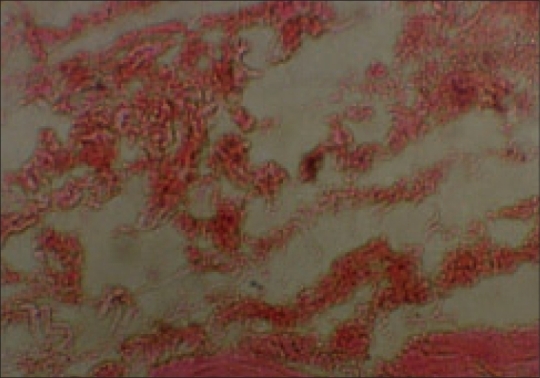
Photograph of histopathological slide of excised contracture band of Case 3 showing chronic inflammatory cells admixed with fibrous tissue

Patients were followed up for 6-17 years (average 9.5 years). All patients achieved correction of deformity and were assessed based on the presence of pain, range of shoulder motion and the strength of deltoid muscle [Figures 4 a,b]. Histopathological study of excised tissue showed features of chronic inflammation admixed with atrophied muscle [Figure 5]. Among complications, hypertrophic scar was seen in one patient (Case 3) operated by longitudinal incision and terminal painful limitation of shoulder motions was seen in another patient (Case 15), possibly from periscapulitis, who had undergone excision of extensive contracture with correction of anterior subluxation of humeral head. This patient with pain was treated subsequently by subacromion (between the overhanging acromion and anterolateral aspect of the humeral head) injection of steroid locally and regained satisfactory function. Another patient (Case 7) though regained almost full arc of shoulder motion, had persistent prominence of vertebral border of scapula.

## DISCUSSION

In present series of deltoid contractures, there was history of intramuscular injection in early childhood in five cases. Moreover, presence of deformity in brother and sister without history of injection may suggest genetic predisposition though it needs genetic studies for validation.

In our series muscular fibrotic contracture (MFC) of the deltoid was found to be prevalent after six years of age as also noted by other workers.^6^ Most of the contractures were segmental full-thickness contractures usually lateral in all and rarely posterolateral (in four patients) in the deltoid muscle. It was more commonly seen on the left side. All the five patients associated with history of intramuscular injection showed lateral contracture bands with secondary posterolateral bands with anterior subluxation of the humeral head. We had no anterior contracture band in any case. Therefore, intramuscular injection may be considered as the incriminating factor resulting in significant deltoid contracture.

In the first case we utilized proximal incision for release (at the level of origin of the deltoid muscle) but switched over to distal incision (near the insertion). We started our study with proximal incision (at the level of origin of the muscle) in the first patient, but rapidly switched over to distal incision (near the insertion of the deltoid muscle). We found that in most of our patients, the contracture bands radiated from a point situated distally near the insertion of the muscle and therefore the former incision near the origin of the deltoid muscle was not considered suitable for adequate excision of all bands. Hence we used a distal incision in the rest of our cases. We used distal longitudinal incision to excise the primary contracture band completely. Four patients who had lateral and posterolateral bands needed distal transverse incision for the division of accessory bands through single incision to avoid extensive muscular damage of which two showed keloid formation over the scar.^6^

In our series, histopathology of excised contracted mass showed evidence of chronic inflammation, along with atrophied muscle and fibrous tissue.

However, all our cases showed satisfactory functional results with disappearance of abduction deformity of shoulder and winging of scapula except in one adult where winging persisted for six months, perhaps due to disuse atrophy of serratus anterior muscle. We had no recurrence of the abduction deformity at the shoulder or winging of scapula in our cases.

Thus, we can conclude from the study that deltoid contracture has the pathology of chronic inflammation with intramuscular injection as the incriminating factor in etiopathogenesis. The condition can be effectively managed by distal release of the deltoid muscle and excision of the muscular fibrotic contracture band using only a distal longitudinal incision for isolated lateral band and distal transverse incision for combined lateral and posterolateral contracted bands.
